# Editorial: *Nucleic Acids Research* annual Web Server Issue in 2015

**DOI:** 10.1093/nar/gkv581

**Published:** 2015-06-27

The 2015 Web Server Issue of Nucleic Acids Research is the 13th in a series of annual special issues dedicated to web-based software resources for analysis and visualization of molecular biology data. It is freely available online under NAR's open access policy. The present issue reports on 97 web servers.

**Topics**. The Web Server Issue places a special emphasis on tools for emerging technologies such as synthetic biology, network and pathway analysis, analysis of high-throughput sequencing data and innovative visualizations. A total of 20 papers deal with these topics. Other major categories include function prediction for genes and proteins (11 papers); DNA and RNA related topics including sequence and structure, alignment, similarity search and motifs (15 papers); non-coding RNA (5 papers); protein structure (13 papers); protein docking and ligand binding (6 papers); and gene and genome analysis (7 papers).

The Issue also contains a special section for papers describing large collections of web services for automated analyses that can be utilized programmatically rather than through manual interaction with a web browser. Six papers fall in this category.

**Acknowledgements**. The Web Server issue would not be possible without the work of the many scientists and programmers who have provided us with outstanding, freely available web resources and the conscientious efforts of literally hundreds of reviewers.

**Figure 1.**
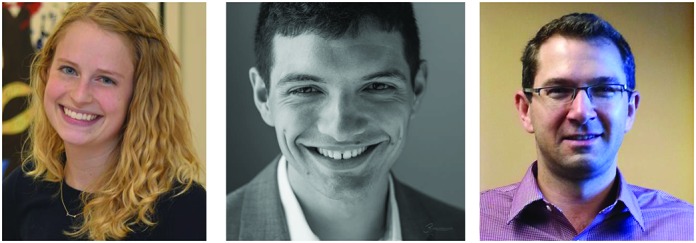
From left: Allyson Byrd and Joe Perez-Rogers are PhD students in the Boston University Bioinformatics Graduate Program. Artem Mamonov is a Research Associate in the BU Department of Biomedical Engineering. They provided outstanding assistance in testing the Web Server proposal websites.

My work was made possible, first, by the dedicated editorial assistance of Fay Oppenheim. Thank you. Thanks also to Allyson Byrd and Joe Perez-Rogers, PhD students in the Boston University Bioinformatics Program, and Artem Mamonov, Research Associate in the Boston University department of Biomedical Engineering, for their outstanding assistance in evaluating the proposal websites (Figure 1). Additional thanks to Martine Bernardes-Silva, Editorial Manager, NAR, and Jennifer Boyd and the staff at Oxford University Press.

**Instructions for Submissions**. To streamline the review process, authors are required to send a one-page summary of their web server to the editor, Dr. Gary Benson (narwbsrv@bu.edu), for pre-approval prior to manuscript submission. For the 2015 issue, 295 summaries were submitted and 118, or 40%, were approved for manuscript submission. Of those approved, 97, or 82%, were ultimately accepted for publication.

Review of a summary includes evaluation of the proposal and extensive testing of web server functionality. The key criteria for pre-approval are high scientific quality, wide interest, the ability to do computations on user-submitted data, and a well-designed, well implemented, and fully functional website. Note that there is a minimum two-year interval before publication in the Web Server issue for web servers, or essentially similar web servers, that have been the subject of a previous publication, including publication in journals other than NAR.

With respect to the website, the following are guidelines for approval.

1. It should have an easy-to-find submission page with a simple mechanism for loading test data and setting test parameters. The preferred method is one-click loading. Additional mechanisms that assist the user in submitting data should be implemented where appropriate, for example, automatic download of a pdb structure file once the user has entered the appropriate identifier.
2. Output should be dynamic and rich in detail. Wherever possible, supporting evidence used in calculations and/or links to external databases containing additional information should be provided. Numerical, textual and visual output should be mixed and any visualization tools that add information or increase the user's understanding should be utilized. Note that output consisting merely of a few numerical values, a static spreadsheet, or a series of files to be opened in other programs will not be approved. Note also that for security reasons, use of FLASH and Java is discouraged.
3. Web servers that do not finish their calculations immediately must implement a mechanism for returning results to the user. Notification by email may be provided as an option, but an alternative that returns a web link at the time of data submission, which the user can then bookmark and access at a later time, is required. This link should ideally report the status of the job (queued, running, finished). Websites that require a guest login will not be approved. Note that uploaded data and the results of analysis for each user must be private and not viewable by other users.
4. The website should be supported by an extensive help section or tutorial that guides the user through the submission process, contains details about input file formats and parameters, and importantly, explains the meaning of the output. Whenever possible, the help pages should link to dynamic output examples similar to those provided by the website. Text and figure help pages, rather than video tutorials, are preferred because they simplify quick look-up.
5. Any proposal for a web server that is *predictive* must include details on validation of predictions from new data not used in training. *N*-fold cross validation methods will not be considered sufficient. Details should include size and composition of the validation data set (number of positive and negative cases), and several measures of predictive performance, including sensitivity, specificity and precision. Proposals are frequently rejected for lack of adequate prediction validation information.
6. Websites not clearly designed to accept and analyze user-submitted data will be rejected. This applies to those established primarily for lookup or exploration in a data set, or serve the function of “data aggregators.” Authors of websites that provide novel data should consider the NAR Database Issue as a possible venue (see the instructions at http://www.oxfordjournals.org/our_journals/nar/for_authors/msprep_database.html).
7. Proposals that describe a new analysis method are generally not appropriate for the Web Server issue because limited space makes adequate method description and validation problematic. Authors of such methods might instead consider sending their manuscript to NAR as a regular computational biology paper (see the instructions for authors at http://www.oxfordjournals.org/our_journals/nar/for_authors/criteria_scope.html#Computational%20Biology).

**Special Emphasis for 2016**. For the 2016 issue, the topics of special emphasis will be tools for synthetic biology design, network and pathway analysis, analysis of high-throughput sequencing data, and innovative visualizations.

**Deadlines for 2016**. Authors wishing to submit manuscripts for the 2016 Web Server issue must submit their one page proposal along with the URL address of the fully functional website to narwbsrv@bu.edu by 31 December 2015. Detailed instructions and requirements are presented at http://www.oxfordjournals.org/nar/for_authors/submission_webserver.html. This information should be consulted before sending in the summary. The deadline for submission of articles is 31 January 2016.

**Requirement for References Links**. Manuscripts submitted for the 2016 issue must format their References section to include active links to electronic versions of the cited papers, including links to PubMed, PubMed Central and a DOI link. Instructions for incorporating these links into the manuscript are presented at http://www.oxfordjournals.org/nar/for_authors/submission_webserver.html.

Gary Benson

Executive Editor

Web Server Issue

Nucleic Acids Research

